# Lord Beaconsfield and His Physicians

**Published:** 1881-05

**Authors:** James I. Tucker

**Affiliations:** Chicago


### ARTICLE III.

LORD BEACONSFIELD AND HIS PHYSICIANS. By DR. JAMES I. TUCKER, Chicago.

"Whereupon Dr. Quain wrote Dr. Kidd, asking him to state his method of treatment." (*Chicago Tribune*, April 17.)

Lord Beaconsfield, late Premier of England, at the age of seventy-seven was taken mortally ill. When such a man falls a victim to disease, his case naturally attracts the attention of the whole civilized world. Whose career was ever more eventful and extraordinary than that of Benjamin Disraeli? Scholar, author, orator, statesman, diplomat. Like his father, the author of that remarkable book, "Curiosities of Literature," his ambition was first in the direction of literature. We have all read his "Vivian Grey," and enjoyed its originality, vivacity and wit. We have perhaps read his "Young Duke," his "Contarini Fleming," interesting to us because its subject is psychological; the "Wondrous Tale of Alvoy," "The Rise of Iskandra," "Henrietta Temple," a love story; "Venetia," in which the characters of Byron and Shelly are so well portrayed; his "Sybil," "Ixion," "Tancred," and last of all, "Endymion," which, like some of the others, is extraordinarily popular because its principal characters are drawn from well-known persons now living. He was a powerful debater, a keen and polished satirist. He became Chancellor of the Exchequer, Member of the Privy Council, leader of the Ministerial party in the House of Commons. Is it strange that such a man, who, by the force of talent, industry and perseverance rose to such an eminence, should attract universal attention now that, while I write, his life is fast ebbing away?*

Therefore, when physicians are called to attend such a man in their professional capacity, their conduct is not only carefully scrutinized, but what they say and do has a deep and lasting influence upon the medical world. Lord Beaconsfield's family

---

* He has since died.

physician had been for years a certain Dr. Kidd. Is it probable that this physician would have obtained and retained the confidence of such a man as Disraeli unless he had possessed certain sterling qualities, both as a man and a medical adviser? But, realizing the grave responsibility of his position, Dr. Kidd sought counsellors. Sir William Jenner, physician to the Queen, refused to go. Why? Because Dr. Kidd was *reputed* to be a homœopathist. Shades of our fathers! What would old Edward Jenner, the illustrious progenitor of this hypercritical and hyperroyal aristocrat have said had he been living at that critical moment? Do we clearly recollect the trials of this great medical reformer? Pupil of John Hunter, he naturally acquired a passion for original investigation. He certainly met with very little sympathy and encouragement from his medical contemporaries, and he was even forbidden, by the medical societies to which he belonged, to indulge in his speculations, under penalty of expulsion! He was accused of "bestializing" his species, by the profession, and his method was pronounced "diabolical" from the pulpit. And yet, now, we all, including Sir William Jenner, physician to the Queen, look upon him as one of the greatest benefactors of mankind. We all vaccinate now. Next, Dr. Quain was called. For the life of him he could not see how his services would be useful, inasmuch as Dr. Kidd was a homœopathic practitioner, and his own method was diametrically opposite. But Dr. Quain sought the advice of Sir George Burrows, ex-President of the College of Physicians. How glad we ought to be that we have in the medical world something akin to a high priesthood or a supreme judiciary, to which we can appeal in time of trouble! So he went—Dr. Quain, of anatomical fame, went—but, wisely, not until he had received from Dr. Kidd an answer to a letter requesting him to state his method of treatment. Dr. Kidd's answer was satisfactory. He said he was not a homœopathist; that he treated his patients with pharmaceutical remedies in pharmacopœial doses, according to the principles of scientific medicine, though he did not feel precluded from using in his practice the *so-called** homœopathic remedies. "Although,"

* The italics are my own.

he writes, "I am a reported homœopath, I desire once for all to disclaim any such party designation. * * * In a very extensive practice, extending over thirty-four years, I have always adopted that course of treatment which my own study and experience have taught me to be most effectual to my patients. Herein I lay claim to having acted as any man does who is not bound by the *trammels of merely mechanical routine.** Like other practitioners, I use the drugs of the British pharmacopœia, but in many cases I have learned from experience that what are called homœopathic remedies may be usefully prescribed. Such remedies I freely use in suitable cases, and according to my own judgment. I do not prescribe infinitesimal doses, nor do I prescribe according to the caprice of my patient."

Is it surprising that Dr. Quain, in view of this explanation—an explanation which Sir William Jenner, descendant of the immortal and persecuted Edward, did not deign to try to obtain—is it surprising, I say, that he was "loud in his praise of Dr. Kidd?" "Moreover, Dr. Quain wrote to Dr. Kidd, asking him to state his method of treatment." The answer was prompt, explicit and manly, and yet Dr. Kidd is still under the ban of professional ostracism, and Dr. Quain has been censured for consulting with him! Is it not time that this narrow antagonism toward those who differ from us in slight degree should be eliminated from the conduct of men who claim to represent a liberal profession and to be actuated by motives of the broadest humanity?

I am glad to learn that one of our brethren, Dr. Fordyce Barker, publicly condemns this antagonism and unreasonable exclusiveness. Let it be understood, once for all, that I have no sympathy whatever with pure homœopathy, or any system which is not based upon the *tolle causam* of Hippocrates, and in its therapeutics is not broad enough (to use the words of Dr. Oliver Wendell Holmes), "to appropriate everything from every source that can be of the slightest use to anybody who is ailing in any way or is like to be ailing from any cause."

It follows, therefore, that any party designation, like allopath,

---

* The italics are my own.

homœopath, isopath, eclectic, hydropath, electric, Thompsonian, medico-physiological, or any other whatsoever, must, in the nature of things, come in violent collision with any enlightened code of ethics, expressed or unexpressed, which is founded upon the Common Sense of the liberal profession of Medicine. A member of this profession can be absolutely *nothing* more or less than PHYSICIAN.

---

MORTALITY IN CHICAGO.—Pop. 503,298. February, 1881, 836; Feb., 1879, 533; Feb., 1880, 738. Greatest mortality, Feb., 1881, was under 10 years of age and between 20 and 30. Tubercular diseases, 97; meningitis, and cerebro-spinal fever, 39; pneumonia, 56; croup and diphtheria, 85; small pox, 33; scarlet fever, 22; infantile convulsions, 63; heart disease, 22; bronchitis, 47; puerperal peritonitis, 22; old age, 26; by violence, 36. These were the most prominent causes; 250 is the total number of deaths referred to zymotic diseases.

SMALL POX continues to prevail throughout the country. Its chief centers are at Philadelphia, which reports between forty and fifty deaths per week; Chicago, where it is next in frequency, eight or nine deaths a week being reported; New York Brooklyn, Pittsburgh, Pennsylvania, and Brownsville, Texas.—*Medical Record.*

ANOTHER CASE OF NEPHRECTOMY in a child, with suppurating and cystic kidney, was performed recently at the Evelina Hospital, by Mr. Morrant Baker. The child was, at last accounts, doing well.—*Ibid.*

BILLROTH'S OPERATION FOR CANCER OF THE STOMACH.—The patient from whom Billroth removed a cancer of the pylorus, by excising part of the stomach, is reported to be nearly well. She takes food by the mouth without trouble.—*Ibid.*

